# Automated machine learning optimizes and accelerates predictive modeling from COVID-19 high throughput datasets

**DOI:** 10.1038/s41598-021-94501-0

**Published:** 2021-07-23

**Authors:** Georgios Papoutsoglou, Makrina Karaglani, Vincenzo Lagani, Naomi Thomson, Oluf Dimitri Røe, Ioannis Tsamardinos, Ekaterini Chatzaki

**Affiliations:** 1 JADBio, Gnosis Data Analysis PC, Science and Technology Park of Crete, N. Plastira 100, Vassilika Vouton, 70013 Heraklion, Crete Greece; 2 Computer Science Department, University of Crete, Voutes Campus, 70013 Heraklion, Crete Greece; 3 Laboratory of Pharmacology, Medical School, Democritus University of Thrace, 68100 Alexandroupolis, Greece; 4 Institute of Chemical Biology, Ilia State University, Kakutsa Cholokashvili Ave 3/5, 0162 Tbilisi, Georgia; 5 Department of Clinical and Molecular Medicine, Norwegian University of Science and Technology, Prinsesse Kristinsgt. 1, 7491 Trondheim, Norway; 6 Clinical Cancer Research Center, Department of Clinical Medicine, Aalborg University Hospital, Hobrovej 18-22, 9100 Aalborg, Denmark; 7 Institute of Agri-Food and Life Sciences, Mediterranean University Research Centre, 71410 Heraklion, Crete Greece

**Keywords:** Infectious diseases, Public health

## Abstract

COVID-19 outbreak brings intense pressure on healthcare systems, with an urgent demand for effective diagnostic, prognostic and therapeutic procedures. Here, we employed Automated Machine Learning (AutoML) to analyze three publicly available high throughput COVID-19 datasets, including proteomic, metabolomic and transcriptomic measurements. Pathway analysis of the selected features was also performed. Analysis of a combined proteomic and metabolomic dataset led to 10 equivalent signatures of two features each, with AUC 0.840 (CI 0.723–0.941) in discriminating severe from non-severe COVID-19 patients. A transcriptomic dataset led to two equivalent signatures of eight features each, with AUC 0.914 (CI 0.865–0.955) in identifying COVID-19 patients from those with a different acute respiratory illness. Another transcriptomic dataset led to two equivalent signatures of nine features each, with AUC 0.967 (CI 0.899–0.996) in identifying COVID-19 patients from virus-free individuals. Signature predictive performance remained high upon validation. Multiple new features emerged and pathway analysis revealed biological relevance by implication in Viral mRNA Translation, Interferon gamma signaling and Innate Immune System pathways. In conclusion, AutoML analysis led to multiple biosignatures of high predictive performance, with reduced features and large choice of alternative predictors. These favorable characteristics are eminent for development of cost-effective assays to contribute to better disease management.

## Introduction

The novel coronavirus SARS-CoV-2 has spread within a few months from the beginning of 2020 to become a world-wide pandemic^1^. At the end of 2020, nearly 100 million people have verified infection, leading to more than 2 million deaths, more than all yearly deaths of lung cancer worldwide. This rapid outbreak brings intense pressure on healthcare systems, with an urgent demand for effective diagnostic, prognostic and therapeutic procedures for COVID-19. Scientists and clinicians are addressing this call with remarkable effort and energy by collecting data and information on diverse domains, as shown by the thousands of articles published on the topic since the beginning of the outbreak^2^. In this endeavor, the pivotal role of Machine Learning (ML) was highlighted early on^3–6^. Accordingly, advanced techniques are currently used for providing useful insights into the pandemic through multivariable prediction models. At the patient level, ML facilitates the classification of viral genomes from sequencing data^7^; it stratifies high risk patients using omics and clinical data^8–10^; and classifies infected and non-infected patients from tomographic images^11,12^ and other imagery data^13,14^. Furthermore, epidemiological studies employ machine learning to predict outbreaks from population migration and epidemiological data^15^, while drug development leverages ML to virtually screen drugs based on data from the viral genomic sequence features^16^. Undoubtedly, ML can greatly add into the fight against the pandemic, as in multiple other healthcare advancements^17^. However, incorporating ML in biomedical classification analysis presents some burden; manual construction of models requires significant statistical and coding knowledge, experience with the choice of algorithms and their tuning hyper-parameters, the feature selection process, and the estimation of performance protocols; furthermore, ML is prone to methodological errors that could lead to overfitting and overestimation. Most importantly, ML requires significant time and effort. Consequently, it is not surprising that despite the immense effort, a recent study concluded that most of the proposed COVID-19 ML models are poorly reported, unreliable when applied to an external group of patients, and misleading because the reported performances are probably optimistic^18^.

**Figure 1:**
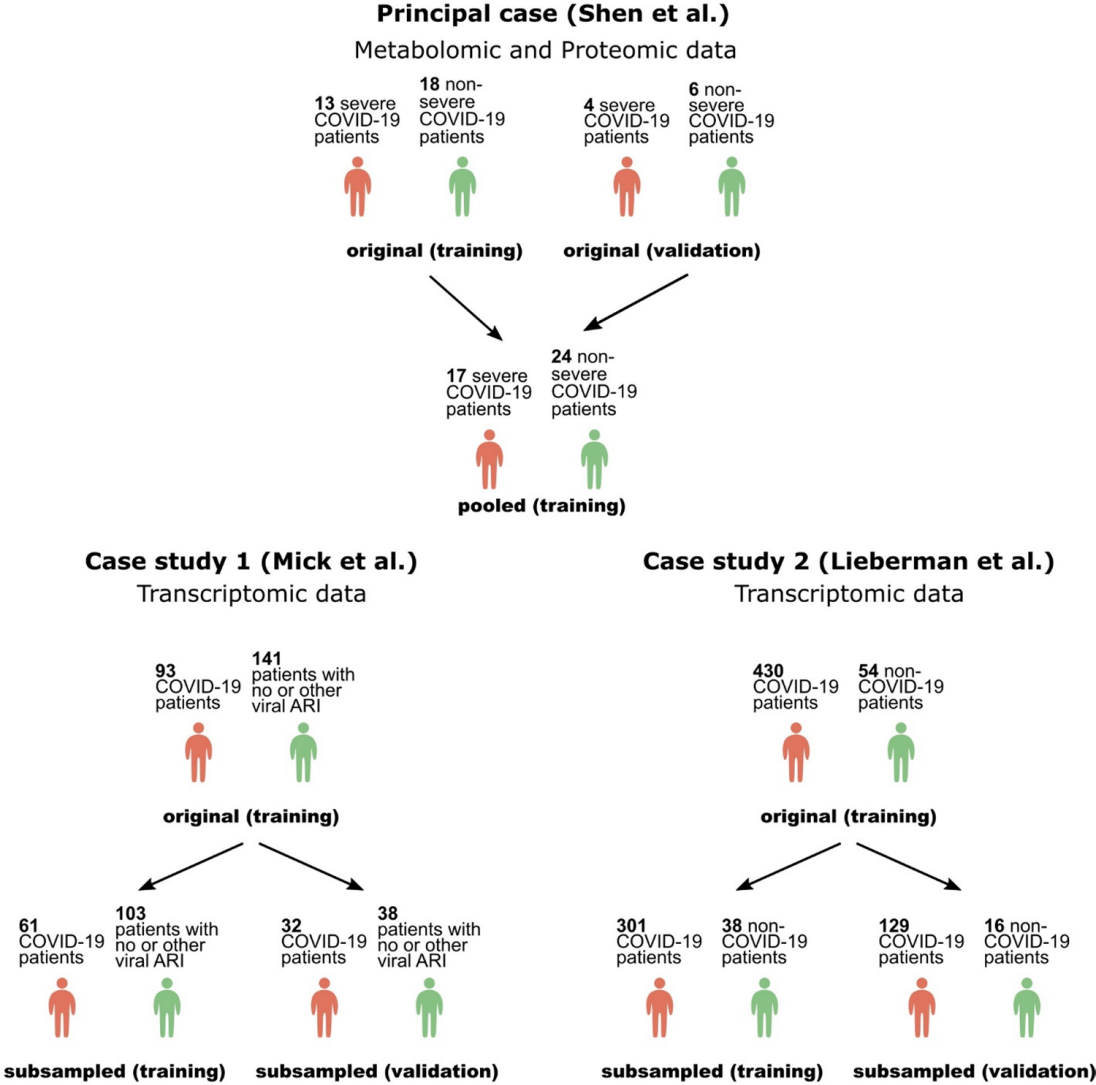
Graphical summary of the COVID-19 datasets re-analyzed in our study. Principal case: Public data from Shen et al. originally include measurements from two independent cohorts, one used for model training and a different one for model validation. The task was to create a model that discriminates between severe and non-severe COVID-19 patients. We also applied JADBio on the pooled data to demonstrate ability to provide reliable estimates when no samples are lost to estimation. Case study 1: The task was to compare COVID-19 patients to those with another viral or no viral acute respiratory illness (ARI). To validate the predictive performance of AutoML, we performed stratified subsampling on the original data: 70% of the samples were assigned for model training and 30% for validation. Case study 2: The task was to identify transcriptional footprints in the responses of COVID-19 patients (individuals with PCR-confirmed SARS-CoV-2 presence). As in case study 1, we validate our AutoML approach on subsampled data.

The most recent solution to alleviate these problems comes from Automated ML (AutoML), which improves the productivity of the model development process in a way that minimizes errors and biases. AutoML automates algorithm selection, hyper-parameter tuning, performance estimation, and result visualization and interpretation. In this way, AutoML tools promise to deliver reliable predictive and diagnostic models that can be interpretable to a non-expert, while drastically increasing the productivity of expert analysts^19^. Towards this end, the recently launched Just Add Data Bio (JADBio) platform (www.jadbio.com). ^20^is an AutoML technology readily applicable to low-sample, high-dimensional biomedical data producing accurate predictive models with their corresponding biosignatures. JADBio is not a black box, as it reports all the combinations of algorithms and their hyper-parameter values tried (see links at S. Table 1), while it offers a free version to replicate results”. To our knowledge, no other AutoML platform can perform feature selection (i.e., identify smalls-size biosignatures) in low-sample, high-dimensional data and report accurate estimates of predictive performance.” JADBio has been extensively validated on 360 omics datasets^20^ demonstrating correct estimates of performance for the produced models on the training data population. By employing this AutoML approach, we have recently produced accurate and validated biosignatures by revisiting high throughput data for Alzheimer’s diagnosis and cancer prognosis, as well as modeling in various other domains^20–27^. Moreover, a COVID-19 model that estimates the probability of viral mutations to produce severe infections has been generated and is currently available in the form of an online resource^28^. In the present study, we were set to join efforts against the COVID-19 pandemic by adopting this AutoML approach onto publicly available –omics datasets. We initially revisited the precious dataset from the study of Shen et al.^9^, combining two moieties of -omics readings, i.e., proteomics and metabolomics, in the same patient cohort, including a full description of 894 proteins and 941 metabolites quantification. By original machine learning analysis, authors produced a Random Forest model composed of 29 serum factors, showing some patient stratification potential. However, the translational value of these findings is questionable as the model presents two severe drawbacks. First, the model includes 22 proteins and 7 metabolites, a rather high feature number to be implemented in a routine laboratory multiplex assay. This reduces clinical applicability when time- and cost-effective solutions are mostly anticipated. Second, when the model was applied on an independently sampled validation cohort of ten subjects, the AUC dropped to 0.875 vs 0.975 estimated in the training cohort using cross-validation based techniques. By employing AutoML, the following challenges were addressed: Could we improve on the predictive power of the models? Can we reduce the number of serum factors needed to be measured without sacrificing performance to develop a cost-effective laboratory test? Can we obtain more accurate training estimates that better reflect the performance anticipated in a real-life setting? Most importantly, can AutoML improve on these aspects in a fully automated mode? To further elaborate on those answers, we also analyzed datasets from two more studies addressing different COVID-19 related clinical questions^29,30^, the latest used here for the first time for classification analysis, in an attempt to maximize predictive performance and accelerate the development of emerging diagnostics/prognostics. A graphical summary of the studies is shown in Fig. 1.

**Table 1:** Predictive performance estimates in terms of AUC reported in the original dataset publication and by JADBio on training and validation sets.

ID	Analysis methodology	Feature selection option (JADBio)	Feature selection algorithm	Modeling algorithm	Training estimate	Validation estimate	#Features selected
1a	Original	–	Random forest	Random forest	0.975	0.875	29
	JADBio	Non-aggressive	Lasso	SVM	0.951 [0.874–1]	0.917	15
	JADBio	Aggressive	SES	Ridge log. regression	0.840 [0.723–0.941]	0.750–1	2 (10 eq.)
2a	Original	–	Lasso	Random forest	0.957 (0.9–1)	0.944	26
	JADBio	Non-aggressive	Lasso	Random forest	0.937 [0.893–0.979]	0.943	24
	JADBio	Aggressive	SES	Random forest	0.918 [0.863–0.959]	0.923	25
3a	JADBio	Non-aggressive	SES	Random forest	0.965 [0.900–1]	0.975–0.981	13 (2 eq.)
	JADBio	Aggressive	SES	Random forest	0.965 [0.900–1]	0.975–0.981	13 (2 eq.)

Numbers in parentheses denote the range of the estimate while the numbers in brackets the 95% confidence intervals. (see Supplementary Table 1 for more results and links to the JADBio platform). The equivalences denote the number of equivalent signatures delivered by JADBio, e.g., “2 (10 eq.)” meaning that JADBio delivered 10 equivalent signatures each containing 2 biomarkers. In the 3a case the same model was generated when either the aggressive or the non-aggressive feature selection option was selected.

The computational experimental methodology was as follows: We applied JADBio on training datasets producing corresponding models, selected feature subsets, and training estimates; then the models were applied on the separate validation sets to obtain testing estimates to compare against. For Shen et al., these correspond to the original cohorts as distributed by the authors. So, this experiment presented us with the opportunity to directly compare JADBio results against Shen. et al. The other two studies from Mick et al. and Lieberman et al. do not provide external validation cohorts. In these cases, we randomly split the original dataset to a training and a validation cohort. To compare against AutoML, we rerun the code originally provided by Mick et al. on the training dataset. For Lieberman et al. no predictive modeling was performed in the original publication to compare against. The results at this stage of the study demonstrated that through this platform overestimation of out-of-sample performance based on the training data was minimized, corroborating several previous works^20^. Thus, we proceeded further, with using the full available data to produce more accurate models, select features, and provide out-of-sample performance estimates. The results were then interpreted biologically.

## Results

### Principal case

We used AutoML on the training cohort data of 13 severe and 18 non-severe COVID-19 patients provided by Shen et al.^9^. An overview of the results is presented in Table 1; links to the complete list of results are included in Supplementary Table 1. Running JADBio with the standard “feature selection” preference on (see Methods), the analysis led to a signature of 15 features with an AUC of 0.917 on the validation dataset; on par with the out-of-sample performance estimation reported on the training dataset, that is, 0.951 (CI 0.874–1). We also ran JADBio with the “aggressive feature selection” preference on (see Methods). AutoML now returned ten equivalent signatures comprising two features each, classifying severe from non-severe disease patients. These signatures lead to equally predictive models up to statistical equivalence. This time, the out-of-sample performance estimate was 0.840 (CI 0.723–0.941). When every equivalent signature was applied on the validation dataset, the validation performance ranged from 0.750 to 1.000 (0.920 on average). This difference is due to the dataset sample size being not large enough to ensure that the difference in achieved performances among the equivalent models is indeed statistically significant. Nevertheless, the range is within or higher than the confidence interval reported in training (Supplementary Table 2).

Regarding the signatures, AutoML suggested two choices for the first predictor, meaning that any of these features can substitute one another: they are informationally equivalent. Then, it suggested five choices for the second predictor (hence, ten signatures and seven biomarkers in total). A total of three out of seven identified features (in all ten signatures) are in common with the 29-feature model described in the original study, as shown in Table 2. Four features (namely Ferritin light chain protein and glycochenodeoxycholate 3-sulfate, adenosine and uracil metabolites) were newly identified by JADBio. Pathway analysis by GeneCards revealed that identified features belong to multiple biological/biochemical pathways, shown in Table 2, including Folate Metabolism, excretion, absorption, and transport of fats and sterols in the intestine and liver and Ferric iron binding. We should note here that feature selection algorithms try to find the smallest set of relevant yet non-redundant markers. This means that some known biomarkers may be filtered out because they were redundant for prediction.

**Table 2:** Features included in the 10 equivalent signatures arising from analyzing the 1639-feature training proteomic and metabolomic data with 13 severe and 18 non-severe COVID-19 patients of the Principal case, and information about their presence in the model reported by Shen et al.

Feature	Protein/Metabolite	Present		Pathway
SAA2 Serum Amyloid A2	Protein	**Yes**		Folate Metabolism
SAA1 Serum Amyloid A1	Protein	**Yes**		Folate Metabolism
Glycochenodeoxycholate 3-sulfate	Metabolite	No		Organic compound
Taurochenodeoxycholic acid 3-sulfate	Metabolite	**Yes**		Excretion, absorption, and transport of fats and sterols in the intestine and liver
Adenosine	Metabolite	No		Component of DNA and RNA
Uracil	Metabolite	No		Component of DNA and RNA
Ferritin light chain	Protein	No		Ferric iron binding

It should be denoted that there is a critical difference between the two approaches, presenting an advantage of the AutoML analysis in terms of translatability of the model: in the original work presented by Shen et al. all 29 biomarkers are needed to construct an optimally performing model; AutoML on the same data built ten alternative models, each containing only two features, and the models can be used interchangeably. In addition, the original predictive model is a non-interpretable Random Forest while the JADBio best predictive algorithm is Ridge Logistic Regression. Because the latter is linear, one can directly interpret the generated model by looking at its analytic formula. For example, the signature of the protein SAA2 and metabolite taurochenodeoxycholic acid 3-sulfate achieves an AUC of 0.958 where, in respect, the estimated log-odds formula is:

ln[P(non severe)/P(severe)] = 0.375 − 0.626*[SAA2] − 0.503*[taurochenodeoxycholic acid 3-sulfate].

**Figure 2:**
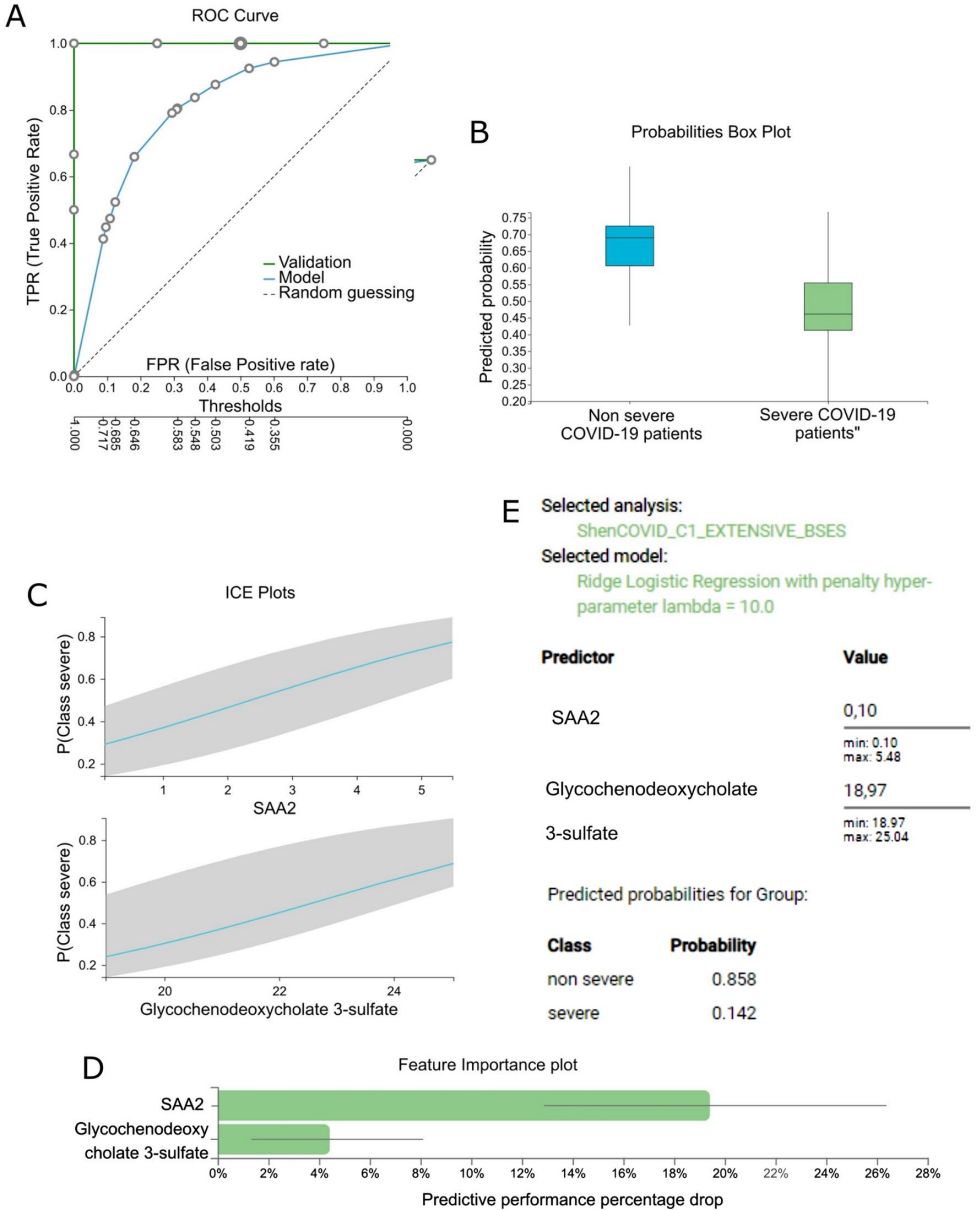
Predictive modeling results of JADBio using aggressive feature selection on the training and validation cohort data of Principal case (Shen et al.). (**A**) ROC plot of the model whose signature consists of SAA2 and glycochenodeoxycholate 3-sulfate showing both the training (blue) and validation (green) ROC curves. (**B**) Box plots of out-of-sample predictions on the training data (i.e., probabilities predicted when the given sample was in the test sets during cross-validation). (**C**) ICE plots of the selected features indicating the average probability predicted for the severe patients’ class given the value of the markers. (**D**) Feature importance for SAA2 and glycochenodeoxycholate 3-sulfate defined as the percentage drop in predictive performance when the feature is removed from the model. The grey lines indicate the 95% confidence intervals. (**E**) JADBio’s interface allows to predict the outcome for single samples, mimicking the application of the predictive model in a clinical setting. Here the (SAA2 and glycochenodeoxycholate 3-sulfate) model is applied on a fictitious patient resulting in predicting the rising of severe symptoms with 0.858 probability.

The negative sign in both coefficients indicates that both features are considered by the model as risk factors. That is, the more their values increase, the less probable is for a patient to be a non-severe case. A second example is the signature of protein SAA2 and metabolite glycochenodeoxycholate 3-sulfate which showed a perfect AUC of 1 through Ridge Logistic Regression. Figure 2 illustrates the respective set of modeling results generated through AutoML. Τhreshold optimization was also possible as follows: the circles on the ROC curve of the model shown in Fig. 2A correspond to different classification thresholds. Each circle reports a different tradeoff between false positive (FPR) and true positive rate (TPR). The user can click on a circle and select the threshold that optimizes the trade-off between FPR and TPR for clinical application. We note that the FPR, TPR and all other metrics (along with confidence intervals) reported in each circle are also adjusted for multiple tries and the “winner’s curse” using the BBC protocol to avoid overestimation (see Methods).

**Figure 3:**
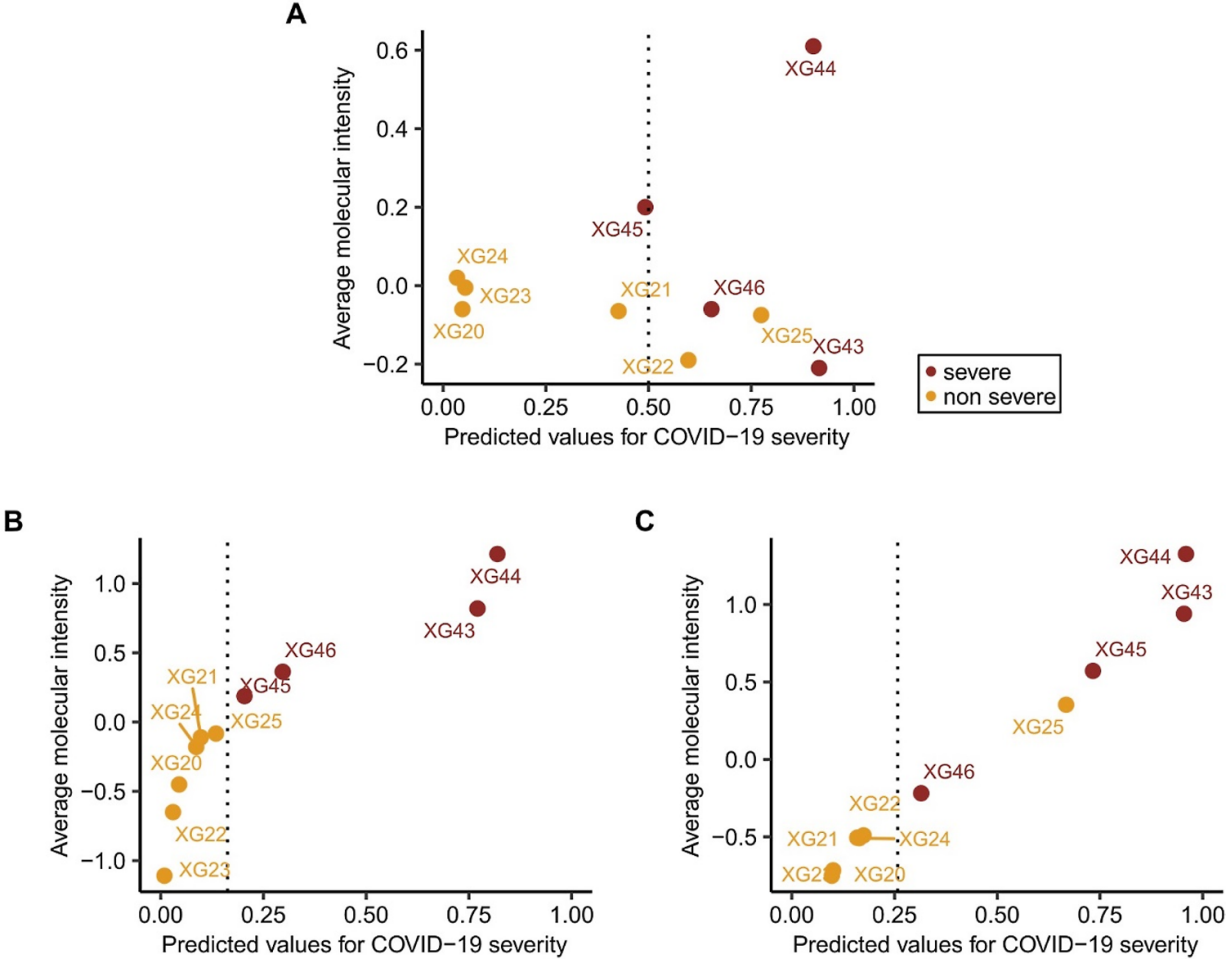
Validation performance results. (**A**) Shen et al.’s model (AUC = 0.875), (**B**) JADBio’s model—SAA2 and glycochenodeoxycholate 3-sulfate (AUC = 1), and (**C**) JADBio’s model—SAA2 and taurochenodeoxycholic acid (AUC = 0.958).

Figure 3 presents the ranking of subjects in the validation cohort according to risk: (A) for the Shen et al., model (B) for the JADBio’s model SAA2 and glycochenodeoxycholate 3-sulfate, and (C) for the JADBio’s model SAA2 and taurochenodeoxycholic acid 3-sulfate. The AUC for the Shen et al. model is 0.875 (21 out of 24 pairs correctly ordered by the model), a significant drop from the 0.95 estimated on the training. Shen et al. classify subjects using a default 0.5 threshold on the probability of becoming a severe COVID-19 case provided by their model. As we see in Fig. 3A this threshold misclassifies three out of ten samples achieving 70% accuracy. Arguably, 70% accuracy is not clinically satisfactory. Shen et al. attribute the low accuracy to comorbidities and previous therapies acting as confounding factors. However, AutoML-built models do rank all patients according to risk almost perfectly. There are only ten subjects in the cohort to allow definitive statements with statistical significance, however, the results corroborate previous extensive evaluations of the platform in terms of correctly estimating out-of-sample performance from the training set alone without the need of a validation set.

Based on this observation, in a next step we performed the AutoML analysis on the complete available samples provided in the original publication in order to produce a more accurate model without sparing samples in validation. Table 3 shows that the training performance estimates with AutoML remain the same, indicating that a separate validation set is indeed not required and confirming that the generated models are not over-optimistic. Moreover, as expected, the increase in the number of samples reduced the uncertainty of estimation and when the aggressive feature selection option was employed, only four out of the ten equivalent signatures remained statistically significantly equivalent. This time there is only one choice for the first predictor, that is, protein SAA2 and two for the second predictor, that is, the metabolites taurochenodeoxycholic acid 3-sulfate and glycochenodeoxycholate 3-sulfate. Individual Conditional Expectation (ICE) plots reveal the role of each of these biomarkers in the prediction of the model; they are all risk factors of severe disease course (see Fig. 2C and Supplementary Fig. 1C). All the links-to-results from the AutoML platform can be found in Supplementary Table 1.

**Table 3:** Comparative evaluation of training performance estimates in terms of AUC on the full set of available data (training and validation).

ID	Analysis methodology	Feature selection option (JADBio)	Feature selection algorithm	Modeling algorithm	Training estimate	#Features selected
1b	Original		Random forest	Random forest	0.975	35
	JADBio	Non-aggressive	Lasso	SVM	0.953 [0.837–1]	24
	JADBio	Aggressive	SES	SVM	0.852 [0.607–1]	2 (2 equiv.)
2b	Original		Lasso	Random forest	0.980 (0.951–1)	26
	JADBio	Non-aggressive	Lasso	Random forest	0.948 [0.908–0.979]	49
	JADBio	Aggressive	SES	Random forest	0.914 [0.865–0.955]	8 (2 equiv.)
3b	JADBio	Non-aggressive	SES	Random forest	0.967 [0.899–0.996]	9 (2 equiv.)
	JADBio	Aggressive	SES	Random forest	0.967 [0.899–0.996]	9 (2 equiv.)

The notation is the same as in Table 2. (see Supplementary Table 1 for more results and links to the JADBio platform). In the case of 3a the same model was generated when either the aggressive or the non-aggressive feature selection option was selected.

### Case study 1

In this study, host/viral metagenomic sequencing profiles of upper airway samples were available from 93 COVID-19 patients, as well as from 141 non-COVID-19 patients experiencing other viral and non-viral acute respiratory illnesses (ARI)^29^. After splitting the samples of the 15,981-feature transcriptomic dataset into training (70%) and validation (30%) subgroups, AutoML analysis produced a single signature and corresponding model with mean AUC performance estimate on the train of 0.937 (CI 0.893–0.979) and 0.918 (CI 0.863–0.959), depending on whether a non-aggressive or an aggressive feature selection approach was employed, respectively (Table 1). The corresponding estimate on the validation set slightly increased, reaching AUC of 0.943 and 0.923 respectively. The methodology provided by Mick et al., produced similarly performing models and corresponding estimates that are statistically indistinguishable with the ones produced here. The benefits of the AutoML application in this case have to do with the fact that analysis was automated requiring no expertise, trying several algorithms and with no need of coding, still reaching optimal results. AutoML was also applied on all available data, i.e., pooled validation and training. When the aggressive feature selection option was employed, two statistically equivalent signatures were built via Classification Random Forests algorithm of eight features each, with mean AUC 0.914 (CI 0.865–0.955) in discriminating the two groups (Table 3). Based on the previous data, we can assume that this significant feature reduction was achieved without compromising model’s performance.

Between the nine features selected by AutoML, four were common with the original model and five new emerged. We should note here that feature selection algorithms that try to find the smallest set of relevant yet non-redundant markers. Pathway analysis shown in Supplementary Table 3, revealed contribution in Interferon gamma signaling and Innate Immune System, Cell signaling during embryo implantation, G-protein signaling Ras family GTPases in kinase cascades (scheme) and Degradation of the extracellular matrix and Epidermal growth factor receptor/EGFR signaling pathways (common ones i.e. IFI27, TRO, TIMP1 and FAM83A) and Microtubule binding and GABA receptor binding, Protein heterodimerization activity, Phospholipase D signaling pathway and Signaling by GPCR, Bacterial infections in CF airways and NF-KappaB Family Pathway and Tryptophan metabolism and Viral mRNA Translation pathways (new ones i.e., GABARAP, TPD52L2, RALGDS, TOLLIP, INMT).

### Case study 2

Gene expression data profiles were collected from nasopharyngeal swabs of 430 individuals with PCR-confirmed SARS-CoV-2 (COVID-19 patients) and 54 non-COVID-19 patients^30^. This 35,787-feature transcriptomic dataset was used here for classification analysis for the first time. Samples were partitioned into a training and validation sets. The training set contained 299 COVID-19 patients and 40 non-COVID-19 patients. AutoML analysis returned the same results with both aggressive and non-aggressive feature selection preference on. Specifically, the analysis returned two equivalent signatures containing 13 features each (shown in Supplementary Table 4). The corresponding model for each signature is a Classification Random Forest; their AUC performances on the training are estimated as 0.965 (CI 0.900–1); in the validation dataset, they exhibited higher AUCs, namely, 0.975 and 0.981, respectively (Table 1), confirming again no initial overestimation. Hence, to avoid losing samples to validation, we performed a second AutoML analysis on the complete data samples. Once again, the preference on feature selection did not make a difference and one model was returned for both aggressive and non-aggressive feature selection. It returned two equivalent signatures of nine features each (shown in Supplementary Table 5), whose corresponding model is a Random Forest having a mean AUC 0.967 (CI 0.899–0.996) (Table 3). A total of nine features were common between built signatures namely CXCL10, PCSK5, ROCK1P1, PMP22, AL022578.1, RPLP1, CXCL9, EFNA4 and PRR7. Pathway analysis revealed involvement in the following pathways: rRNA processing in the nucleus and cytosol and Viral mRNA Translation, Signaling by GPCR and PEDF Induced Signaling, NMDA receptor-mediated excitotoxicity, Signaling by GPCR and PEDF Induced Signaling, Lipoprotein metabolism and Signaling by GPCR, Guidance Cues and Growth Cone Motility and Ras signaling pathway, GABAergic synapse and Respiratory electron transport and Neural Crest Differentiation and a6b1 and a6b4 Integrin signaling. As in the original study of Lieberman et al., no predictive modelling has been performed, so there is no methodology or model to compare against. Our analysis is a first discovery and validation analysis of this dataset.

## Discussion

In this study, we applied AutoML in order to obtain accurate diagnostic/predictive models for COVID-19, using available archived proteomics, metabolomics and transcriptomics datasets. In an automated way and with minimal expert effort, we improved or confirmed the predictive signatures obtained in the original publications^9,29^ and discovered novel signatures^30^. Quite importantly for clinical application, biosignatures built here were often reduced in feature number, without compromising in predictive performance and there were multiple equivalent options in parameters included providing choices to the designers of diagnostic assays. Furthermore, the respective predictive performance estimates accurately reflected the performance obtained on the validation sets, allowing secondary analysis of the entire dataset to deliver several highly diagnostic/prognostic biosignatures of minimal feature size from different types of COVID-19 data.

In our principal case, we revisited the data published by Shen et al.^9^. Authors produced proteomic and metabolomic profiles from sera of COVID-19 patients and comparisons were made between severe and non-severe disease courses. Multiple proteins and metabolites were found to be differentially expressed, and pathway analysis highlighted metabolic and immune dysregulation in seriously ill COVID-19 patients. Moreover, standard machine learning modeling was employed to reveal stratification signatures. Using a Random Forest classification model on the training dataset, the authors computed Feature Importance and arbitrarily selected the top 29, including 22 proteins and seven metabolites. In turn, they built a predictive model using the same random forest on the reduced training dataset that reached an AUC of 0.957 in classifying severe vs non-severe COVID-19 patients. However, upon validation in independent datasets, the model showed rather poorer performance (albeit, only ten samples were available for validation). At a closer look on their machine learning approach, one can comment that there is room for improvements (e.g., only one algorithm is tried, possible returns of redundant features by Random Forest’s Feature Importance, it is likely that their estimate of performance is too optimistic as shown in validation).

Our AutoML analysis of the same dataset was able to effectively select just a handful of serum markers for inclusion in the predictive models without a drop in predictive performance. Specifically, it produced ten equivalent signatures, all containing a significantly reduced feature number, in fact down to two. This size would present a major advantage upon application in a disease progression prognostic test, as it provides choices for a feasible solution for multiplexing in a simple laboratory test that can be implemented in a standard equipped laboratory. Most importantly, some of the equivalent signatures contained only proteomic features, providing the choice for one type of measurement and improving assay cost-effectiveness. The AUCs of these models, although still high, were sometimes lower than those reported for the Shen group’s model. However, the performance of our models was stable, in fact better upon validation, showing successful addressing of overestimations and overfitting and supporting accurate prediction in a clinically relevant application. Clinical confounding conditions or pharmacotherapy used to explain non-accurate predictions of the original classifier did not seem to present a problem with the AutoML signatures. Overall, by applying AutoML, the two most important limitations of the original analysis in terms of translatability were raised.

Moving forward, we applied our AutoML approach of the pooled data samples to reduce the number of signatures to only two. These include three features, a protein and two metabolites, all of which are identified as risk factors of severe disease course. From a pathophysiology point of view, the biomarkers SAA2 and taurochenodeoxycholic acid 3-sulfate are common to the Shen et al. 29-feature model; while, the second metabolite (glycochenodeoxycholate 3-sulfate), emerged by our analysis as a critical classifying parameter. SAA2 (Serum Amyloid A2) is a variant of Serum Amyloid A (SAA); a protein that participates in the metabolism of folate and is known to present increased serum levels upon acute bacterial or viral infections^31^. In fact, it has been found that as COVID-19 progresses SAA gradually increases signifying poor prognosis^32^. Intriguingly, this is on par with our AutoML findings in that SAA2 can be used as a predictor of the severe prognosis in patients with COVID-19. On the other hand, taurochenodeoxycholic acid 3-sulfate and glycochenodeoxycholate 3-sulfate are bile acid derivatives. As indicated by Shen et al., the elevation of bile acid derivatives could suggest suppressed hepatic detoxification. Furthermore, bile acids have been proposed to possess anti-inflammatory properties that may prove beneficial in curbing the “cytokine storm” believed to be involved in the pathogenicity of the SARS-CoV-2 virus^33^. The fact that the deployment of our approach led to the surfacing of glycochenodeoxycholate-3-sulfate, highlights the offer of secondary data extrapolation and information digging by machine learning. Further study in hypothesis-driven approaches focusing on its role in coronaviral infections would certainly be worthy of attention.

To examine the efficacy of our AutoML approach in generating highly predictive models in COVID-19 related studies, we used two more publicly available datasets that focus on different clinical questions. The first (case study 1) was the study by Mick et al.^29^ where upper airway samples from COVID-19 patients were compared to those from other viral or non-viral respiratory illnesses (ARI). Gene expression profiles (transcriptomic data) from host/viral metagenomic sequencing were acquired. Using a 15,981-feature dataset and employing a combination of lasso regularized regression and random forest, authors presented a 26-feature classification signature which reached an AUC of 0.980 (range 0.951–1) in identifying COVID-19 from patients with other viral and non-viral ARI, as estimated by fivefold cross validation. In order to facilitate the practical incorporation of their classifier into a clinical PCR assay, they arbitrarily adopted more restrictive regression penalties and identified a 10-feature and a 3-feature classifier. JADBio analysis of the same dataset led to two equivalent signatures of eight features each with an AUC of 0.914 and multiple new features emerged in our study to contribute to the classifying power. Pathway analysis revealed multiple related functions of the new features, such as Viral mRNA Translation (INMT), GABA receptor binding (GABARAP), Bacterial infections in CF airways (TOLLIP), signaling (RALGDS) and others. The GABARAP is an important factor in cellular autophagy and has been identified as a critical component in the mechanism allowing the HIV1/AIDS virus to propagate in cells^34^. These findings seem highly relevant biologically and are novel, showing the potential of the AutoML method to unravel new players in the pathological process. Among the common features, IFI27 was found to be up-regulated in the COVID-19 patients compared to the controls in the study of Huang et al.^35^ while the FAM83A has been found to be differentially expressed between SARS-CoV-2 infected patients and virus-free individuals^36^.

Finally, in our case study 2, we elaborated on the dataset of Lieberman et al.^30^, including gene expression profiles (transcriptomic data) examined in nasopharyngeal swabs from COVID-19 patients (individuals with PCR-confirmed SARS-CoV-2 presence) and non-COVID-19 patients. In the original publication, the authors thoroughly report that host responses to SARS-CoV-2 are dependent on viral load and infection time course, with observed differences due to age and sex that may contribute to disease severity. These data were not used for predictive modeling in the framework of the published study and no predictive signatures are presented. Our analysis is therefore the first discovery and validation analysis of this dataset. Using AutoML on the whole data set, we were able to deliver two equivalent signatures of nine features that reached an AUC 0.967, whereas in the automatically randomly split dataset, two equivalent signatures of 13 features emerged, with AUC 0.965 that was stable upon validation. Pathway analysis revealed multiple related functions, such as Viral mRNA Translation (RPLP1), signaling (CXCL9, CXCL10, PCSK5, EFNA4, IFIT1) and others. The proteins encoded by the *CXCL9 and CXCL10* genes are highly significant players in the so-called “cytokine storm” produced by the corona-virus infection^37^. In addition, PCSK5, ROCK1P1, RPLP1, and NDUFV1 were found to be differentially expressed between SARS-CoV-2 infected patients and non-infected individuals in the study of Vastrad et al.^36^. These data confirm that features selected by AutoML are biologically relevant, some of them already confirmed, others emerging here for the first time. It is important that their contribution to the pathological process is further elucidated in order to fully understand the disease course.

The AutoML approach used here presented several advantages. A. It significantly enhances productivity, which is particularly important in emergency situations, as with COVID-19, where data need to be analyzed immediately to inform public policy. The execution time of the analyses presented ranges between 8–73 min, while the human effort required takes a few clicks. B. It allows democratization to life scientists, as it can be performed by non-expert analysts through a graphical user interface, meaning that a medical doctor has immediate access to the results without relying on analysts for modeling and interpretation. C. It guarantees correctness in the sense that the produced performance estimates follow best-practices in the field and are not overestimated, avoiding common methodological pitfalls that are frequently encountered in ML analyses of omics data. For example, a common issue that leads to inflated performance estimates is the pre-filtering of features by accounting label information on the complete dataset (e.g., by differential expression) and then cross validating only the modeling algorithm on the same data (see Methods). This approach can significantly overestimate performance of the final model. D. It guarantees optimization, as the returned models are competitive, in terms of predictive performance, against human expert models. In addition, the returned signatures (selected feature subsets) are often smaller than the ones returned by humanly crafted code scripts. This is of particular significance if the returned models are directed to be translated to benchtop assays for clinical use. Of course, the human data scientist is still required to collect and prepare the data, and most importantly, formulate a science problem or working hypothesis into a machine learning problem. E. It allows data and model provenance, as replicability and reproducibility of results is possible. This addresses the problem of reproducing the ML results of a published paper due to changes in the code over time. In AutoML, in functional results links (like those presented here) the code version used is recorded and could be reverted back to reproduce an old analysis.

Feature construction methods could present an alternative to substitute feature selection methods. Unfortunately, however, such methods typically come at the cost of losing interpretability and requiring all original features to be computed. They are quite useful when predictive performance is the sole goal, but less so when the purpose is to design a diagnostic assay that requires the measurements of the least number of markers.

Limitations of the present study is the small number of datasets and the subjects included. As more -omics readings will become available from COVID-19 related study groups, the same approach might deliver better classifying models. In addition, the partitioning to training and validation of the available datasets was performed only once. Ideally, one would repeat the process several times and report the average behavior. However, this approach would make the exposition of the biological results less clear. Finally, a major limitation is the lack of external validation sets for the identified signatures and models. Τhe temporal evolution of the disease and the treatment of the time dimension of the pandemic would be another aspect that was currently considered outside the scope of this paper. However, future work could use recurrent neural networks or some similar methodology to incorporate time information into the analyses.

In conclusion, AutoML is a new reality in biomedicine that promises to democratize data analysis to non-experts, drastically increase productivity, improve replicability, facilitate the interpretation of results and shield against common methodological analysis pitfalls such as overfitting^20^. With minimal human effort, it allows extracting maximum information from laborious and expensive measurements of precious biomedical samples, thus enabling personalized clinical decisions and improved disease management. Along these lines, we joined the battle against the COVID-19 pandemic. Earlier produced biosignatures based on machine learning pipelines contain large numbers of predictors, hampering their clinical adaptation. Moreover, their performance often drops significantly when validated in independent groups, which is expected as sample numbers are often inevitably low. With the bioinformatic tools used here, we were able to confirm superiority of this automated and validated approach in terms of accurate performance prediction, effective feature selection to reduced numbers, large choice of alternative predictors, and maximum data extrapolation to biologically relevant indicators. The predictive models presented here are readily available to the scientific community for further development to cost-effective assays to contribute to patient stratification, better disease management and reduction of COVID-19 mortality rates. Our results also highlight the importance of revisiting precious and well-built datasets for maximal conclusion extraction from a given experimental observation.

## Methods

### AutoML analysis using JADBio

A detailed description of JADBio's pipeline is presented in Tsamardinos et al. publication^20^. The dataset to analyze is input to JADBio as a 2D matrix with the rows corresponding to samples and the columns corresponding to measured features (a.k.a. biomarkers, variables, measured quantities, predictors) and a defined outcome of interest. JADBio accepts dichotomous (binary classification), nominal (multi-class classification), continues (regression), and time-to-event (e.g., survival analysis) type of outcomes. Hence, COVID-19 related outcomes may be the severity of the disease, the time of death (survival analysis), or response to therapy. Then, based on the size and type of the input data and the user preferences (e.g., the computation time to allow for the analysis), JADBio fully automatically analyzes the dataset. An AI Decision Support System decides which algorithms for preprocessing, imputation of missing values, feature selection, and modeling to combine, along with their hyper-parameter values, in an effort to produce the optimal model. Essentially, each choice is a unique ML pipeline that takes the dataset and returns a predictive model; we call it a configuration. The number of configurations to try typically ranges between a few tens to a few thousand. Finally, the AI system decides on the hyper-parameter tuning and performance estimation protocol, i.e., how to estimate the performance of each configuration and select the winner to produce the final predictive model. For small sample datasets (or highly imbalanced ones), the performance estimation protocol is a stratified, *n* repeated, *K*-fold Cross Validation (CV) which has been shown to lead to smaller variance of estimation of performance and higher probability of selecting the optimal configuration^38^. For large datasets JADBio may decide to use a simple Hold-Out. Notice that configurations are cross-validated as atoms, i.e., all the combined algorithmic steps are simultaneously cross-validated as one. This avoids serious methodological errors, such as performing feature selection on the whole dataset first, and then estimating performance by cross-validating only the modeling algorithm (see [^39^, page 245] for an eye-opening experiment of the severity of this type of error). Once all decisions are made, JADBio searches in the space of admissible configurations to identify the one leading to the optimal performance^20^. The final model and final selection of features are produced by applying the winning configuration on the full dataset: on average this will lead to the optimal model out of all tries. Thus, JADBio does not lose samples to estimation. To estimate the performance of the final model JADBio uses the Bootstrap Bias Correction estimation method or BBC^38^. BBC is conceptually equivalent to adjusting *p-*values in hypothesis testing for the fact that many hypotheses have been tested; similarly, BBC adjusts prediction performances for the fact that many configurations (combinations of algorithms) have been tried. JADBio’s performance estimation has been validated in large computational experiments^20^: on average it is conservative. Hence, JADBio removes the need for an external validation set, provided it is applied on populations with the same distribution as the training data (JADBio does not remove the need for externally validating the presence of other factors that may affect performance such as systematic biases, batch effects, data distribution drifts, and others). JADBio provides an option for “aggressive feature selection” preference. Aggressive feature selection tries feature selection algorithms that on average return fewer selected features at a possible expense of predictive performance. The feature selection algorithms may also return multiple selected feature subsets (signatures) that lead to equally predictive models, up to statistical equivalence based on the training set. Regarding the specific algorithms tried, for classification tasks JADBio employs Lasso^40^ and Statistical Equivalent Signatures or SES^41^ for feature selection. Such algorithms do not only remove irrelevant features, as differential expression analysis does, but also features that are redundant given the selected ones, i.e., they do not carry any informational added value for prediction. Hence, feature selection considers features in combination, while differential expression analysis does not. In addition, we would like to note that SES performs multiple feature selection and not single feature selection as Lasso. Specifically, as its name suggests, SES reports multiple feature selection subsets that lead to equally predictive models (up to statistical equivalence). This is important for providing choices to the designer of diagnostic assays and laboratory tests to select to measure the markers that are cost-effective to measure reliably. For modeling, JADBio tries Decision Trees, Ridge Logistic Regression, Random Forests, (linear, polynomial, and RBF) Support Vector Machines, as well as the baseline algorithm that classifies to the most prevalent class. However, we do note that this list is only indicative, as we constantly keep enriching the algorithmic arsenal of JADBio. All of the above are transparent to the user who is not required to make any analysis decisions. JADBio outputs (i) the (bio)signature, i.e., the minimal subset of features that ensures maximal predictive power, (ii) the optimal predictive model associated with the selected biosignature, (iii) estimates of predictive performance of the final model along with its confidence interval, (iv) numerous other visuals to interpret results. These include graphs that explain the role of the features in the optimal model [called ICE plots^42^], estimates of the added value of each feature in the model, residual plots, samples that are identified as “hard to predict”, and others.

Threshold selection and optimization for clinical application is an important issue. Using a default 0.5 threshold on the probability is problematic: (i) the probabilities output by Random Forests and similar machine learning models are not trustworthy. They indicate the relative risk of the patient but they are not calibrated^43^. (ii) A 0.5 threshold assumes that the cost of false negative predictions is equal to the cost of false positive predictions. Obviously, this is not the case for COVID-19 severe cases: falsely predicting patients as ‘non-severe’ critically affects their survival while falsely 'severe' considered patients may just take stronger treatments and/or make unnecessary use of medical resources. This means that, for clinical applications, the classification threshold needs to be optimized. JADBio facilitates threshold optimization as follows: the circles on the ROC curve of the model correspond to different classification thresholds. Each circle reports a different tradeoff between false positive (FPR) and true positive rate (TPR). The user can click on a circle and select the threshold that optimizes the trade-off between FPR and TPR for clinical application. We note that the FPR, TPR and all other metrics (along with confidence intervals) reported in each circle are also adjusted for multiple tries and the “winner’s curse” using the BBC protocol to avoid overestimation.

To avoid comparing predictive performance between training and test sets with different class balance, we employ only performance metrics that are independent and invariant to the class distribution (balancing), ie the Area Under the ROC Curve (AUC) and the Average Precision (equivalent to the area under the precision-recall curve). For the purpose of the current analysis, all comparisons employ the AUC metric.

### Datasets

*Principal Case*: Proteomic and metabolomic profiles from sera of COVID-19 patients with severe and non-severe disease were retrieved from Shen et al. publication^9^. Three datasets were downloaded: a training dataset (C1 Training) of 13 severe and 18 non-severe COVID-19 patients containing 1638 features, a validation dataset (C2 Validation) of four severe and six non-severe COVID-19 patients containing 1589 features and another validation dataset (C3 Validation) of 12 severe and seven non-severe COVID-19 patients containing 29 targeted features. Although the authors kindly agreed to provide all data and information, we could not validate our models in C3 as the values were obtained through a different technology (targeted metabolomics). In any case, the features measured in C3 only partially overlapped with those selected through AutoML, so such validation would not be relevant.

*Case Study 1*: Gene expression profiles from host/viral metagenomic sequencing of upper airway samples of COVID-19 patients were compared with patients with other viral and non-viral acute respiratory illnesses (ARIs). There were 93 COVID-19 patients, 100 patients with other viral ARI and 41 patients with non-viral ARI that were retrieved from Mick et al.’s publication^29^ and the GSE156063 dataset from GEO database. In specific, three datasets were used: A. a training dataset of 93 COVID-19 patients and 141 patients with ARIs (viral and non-viral) of 15,981 features, B. a training dataset of 93 COVID-19 patients and 100 patients with other viral ARI of 15,981 features and C. another training dataset of 93 COVID-19 patients and 41 patients with non-viral ARI of 15,981 features.

*Case Study 2*: Nasopharyngeal swab samples analyzed with RNA-sequencing from COVID-19 versus non-COVID-19 patients were compared. The dataset contained 35,787 features from nasopharyngeal swab samples of 430 individuals with PCR-confirmed SARS-CoV-2 presence (COVID-19 patients) and 54 non-COVID-19 patients (negative controls) that were retrieved from Lieberman et al.’s publication^30^ and the GSE152075 dataset from GEO database.

### Data preprocessing

The C1 and C2 cohort data of Shen et al. were used as they were publicly deposited. The raw Mick et al. data were preprocessed as in the original publication (variance stabilization) using the code provided by the authors. For the Lieberman et al. dataset, we followed the data preprocessing performed in the original publication and filtered out from all analyses those genes whose average counts were 1 or 0.

### Pathway analysis

The biological involvement and related pathways of identified features were searched using the GeneCards—The Human gene database tool (https://www.genecards.org/).

### Results and Software availability

The complete analysis results for each dataset such as the configurations tried, the configurations selected to produce the final model, the estimation protocol and the out-of-sample predictions of the cross-validations are reported via unique links in Supplementary Table 1. These results could be employed to compare JADBio against any other methodology, architecture, or algorithm on the same datasets. JADBio is available as SaaS platform at www.jadbio.com where a free trial version is offered. In addition, a free research license is guaranteed for researchers that wish to reproduce results or compare against the current version of JADBio (restrictions apply). Researchers can apply for this license by sending a request on the platform’s webpage. JADBio mainly uses in-house implementations of individual, widely accepted, algorithms (e.g., Decision Trees, Random Forests, etc.) and of unique algorithms namely the SES multiple feature selection algorithm, and the BBC-CV for adjusting CV performance for multiple testing. Open-source implementations of the latter two are available at the MXM R Package^41^. Supplementary Table 6 reports all individual algorithms employed by JADBio.

## Supplementary Information

Supplementary Information.

## Data Availability

All datasets used in the paper are publicly available. Original data for datasets analyzed can be found in the following publications: Lieberman et al.^28^; Mick et al.^27^ and Shen et al.^9^.
