# Supplementary material for: Automated machine learning optimizes and accelerates predictive modeling from COVID-19 high throughput datasets
Source: Sci Rep. 2021 Jul 23;11:15107. doi: 10.1038/s41598-021-94501-0 (PMC8302755; doi:10.1038/s41598-021-94501-0)
Supplement: Supplementary file 1 — Supplementary Information. [file 41598_2021_94501_MOESM1_ESM.docx]

**Automated Machine Learning optimizes and accelerates predictive modeling from COVID-19 high throughput datasets.**

Georgios Papoutsoglou^$^, Makrina Karaglani^$^, Vincenzo Lagani, Naomi Thomson, Oluf Dimitri Røe, Ioannis Tsamardinos^#^, Ekaterini Chatzaki^#^

**Supplementary information**

**Supplementary Figure 1** *Predictive modeling results of JADBio using aggressive feature selection on the training and validation cohort data of Principal case (Shen et al.).* (A) ROC plot of the model whose signature consists of SAA2 and Taurochenodeoxycholic acid 3-sulfate showing both the training (blue) and validation (green) ROC curves (AUC = 0.958). (B) ICE plots of the selected features indicating the average probability predicted for the severe patients’ class given the value of the markers. (C) Feature importance for SAA2 and Taurochenodeoxycholic acid 3-sulfate defined as the percentage drop in predictive performance when the feature is removed from the model. The grey lines indicate the 95% confidence intervals. ​

**Supplementary Table 1** Predictive performance estimates in terms of AUC reported in the original dataset publication (blank), by JADBio on the full set of available data (i.e., no samples are lost to estimation) and on training and validation sets (blue). Numbers in parentheses denote the range of the estimate while the numbers in brackets the 95% confidence intervals. The equivalences denote the number of equivalent signatures found by JADBio, e.g., “2 (10 equiv.)” means that JADBio discovered 10 equivalent signatures each containing 2 biomarkers. Each link to the JADBio platform leads to a report with the complete list of AutoML results. In the case of Lieberman et al., the same model was generated when either the aggressive or the non-aggressive feature selection option was selected. JADBio does not overestimate, no samples to estimation.

**Supplementary Table 2** List of the ten equivalent signatures that were estimated using AutoML under aggressive feature selection, on the data of the Principal case study.

**Supplementary Table 3** The nine features of the two 8-feature signatures of the original 15981-feature transcriptomic data between COVID-19 patients and non-COVID-19 patients of Case study 1, including information about their presence in Mick et al models.

**Supplementary Table 4** The 14 features of the two 13-feature signatures of the subsampled 35787-feature transcriptomic data between COVID-19 patients and non-COVID-19 patients of Case study 2. (N/A: Non-Available)

**Supplementary Table 5** The ten features of the two 9-feature signatures of the original 35787-feature transcriptomic data between COVID-19 patients and non-COVID-19 patients of Case study 2. (N/A: Non- Available)

**Supplementary Table 6** Algorithms used for AutoML in JADBio v1.2.16.

**Supplementary Table 1** Predictive performance estimates in terms of AUC reported in the original dataset publication (black), by JADBio on the full set of available data (i.e., no samples are lost to estimation) and on training and validation sets (blue). Numbers in parentheses denote the range of the estimate while the numbers in brackets the 95% confidence intervals. The equivalences denote the number of equivalent signatures found by JADBio, e.g., “2 (10 equiv.)” means that JADBio discovered 10 equivalent signatures each containing 2 biomarkers. Each link to the JADBio platform leads to a report with the complete list of AutoML results. In the case of Lieberman et al., the same model was generated when either the aggressive or the non-aggressive feature selection option was selected. JADbio does not overestimate, no samples to estimation.

| **id** | **Analysis Methodology** | **FS option (JADBio)** | **FS algorithm** | **Modeling algorithm** | **#Configuatations tried** | **#Models trained** | **Execution time** | **Training estimate** | **Validation estimate** | **#Features selected** | **Link-to-results to the JADBio platform** |
| --- | --- | --- | --- | --- | --- | --- | --- | --- | --- | --- | --- |
| **1a** | Original |  | random forest | random forest | 1 | 1000 |  | 0.975 (max) | 0.875 | 29 |  |
|  | JADBio | non- aggressive | lasso | SVM | 3017 | 162918 | 1h2m | 0.951 [0.874 - 1] | 0.917 | 15 | <https://app.jadbio.com/share/68570d0c-d83b-4fa0-913b-945baf7481af> |
|  | .. | aggressive | SES | ridge log. regression | 1393 | 75222 | 29min | 0.840 [0.723 – 0.941] | 0.750 - 1.000 | 2 (10 equiv.) | <https://app.jadbio.com/share/28e07dea-9b03-4222-b4c4-b13c4e9afd76> |
| **1b** | Original |  | random forest | random forest | 1 | 1000 |  | 0.975 | - | 35 |  |
|  | .. | non- aggressive | lasso | SVM | 3017 | 120680 | 34min | 0.953 [0.837 - 1] | - | 24 | <https://app.jadbio.com/share/a600a0e3-b38d-4fc8-aaee-c71482bcedbb> |
|  | .. | aggressive | SES | SVM | 1393 | 22288 | 5min | 0.852 [0.607 – 1] | - | 2 (2 equiv.) | <https://app.jadbio.com/share/9a8b7b5a-4c1f-472e-8ed2-882f510522db> |
| **2a** | Original |  | lasso | random forest | 5 | 25 |  | 0.957 (0.9 - 1) | 0.944 | 26 |  |
|  | JADBio | non- aggressive | lasso | random forest | 3017 | 60340 | 26min | 0.937 [0.883 - 0.979] | 0.943 | 24 | <https://app.jadbio.com/share/aa688aa1-e485-4337-bb16-53988c6f0ec9> |
|  | .. | aggressive | SES | random forest | 1393 | 41790 | 24min | 0.918 [0.863 - 0.959] | 0.923 | 25 | <https://app.jadbio.com/share/2203e5a7-d946-43ea-a7cb-a953a7907993> |
| **2b** | Original |  | lasso | random forest | 5 | 25 |  | 0.98 (0.951 - 1) | - | 26 |  |
|  | JADBio | non- aggressive | lasso | random forest | 3017 | 60340 | 41min | 0.948 [0.908 - 0.979] | - | 49 | <https://app.jadbio.com/share/bb1a006f-5eb4-4563-b790-caa15f240e06> |
|  | .. | aggressive | SES | random forest | 1393 | 27860 | 22min | 0.914 [0.865 - 0.955] | - | 8 (2 equiv.) | <https://app.jadbio.com/share/426c5566-b5eb-4cc7-96c0-39c0dedb2708> |
| **3a** | JADBio | non- aggressive | SES | random forest | 3017 | 30170 | 15min | 0.965 [0.900 - 1] | 0.975 - 0.981 | 13 (2 equiv.) | <https://app.jadbio.com/share/43ff7497-e770-4c95-a38b-95d857fd0a25> |
|  | .. | aggressive | SES | random forest | 3017 | 30170 | 15min | 0.965 [0.900 - 1] | 0.975 - 0.981 | 13 (2 equiv.) | <https://app.jadbio.com/share/43ff7497-e770-4c95-a38b-95d857fd0a25> |
| **3b** | JADBio | non- aggressive | SES | random forest | 3017 | 30170 | 24min | 0.967 [0.899 - 0.996] | - | 9 (2 equiv.) | <https://app.jadbio.com/share/3d6f6c36-5ca9-4232-8b91-82fa307ced54> |
|  | .. | aggressive | SES | random forest | 3017 | 30170 | 24min | 0.967 [0.899 - 0.996] | - | 9 (2 equiv.) | https://app.jadbio.com/share/3d6f6c36-5ca9-4232-8b91-82fa307ced54 |

**Supplementary Table 2** List of the ten equivalent signatures that were estimated using AutoML under aggressive feature selection, on the data of the Principal case study.

| **Signature #** | **Feature 1** | **Feature 2** | **Validation AUC** |
| --- | --- | --- | --- |
| 1 | SAA2 | taurochenodeoxycholic acid 3-sulfate | 0.958 |
| 2 | SAA2 | uracil | 0.792 |
| 3 | SAA2 | glycochenodeoxycholate 3-sulfate | 1 |
| 4 | SAA2 | adenosine | 1 |
| 5 | SAA2 | ferritin | 0.833 |
| 6 | SAA1 | taurochenodeoxycholic acid 3-sulfate | 1 |
| 7 | SAA1 | uracil | 0.75 |
| 8 | SAA1 | glycochenodeoxycholate 3-sulfate | 1 |
| 9 | SAA1 | adenosine | 1 |
| 10 | SAA1 | ferritin | 0.875 |

**Supplementary Table 3** The nine features of the two 8-feature signatures of the original 15981-feature transcriptomic data between COVID-19 patients and non-COVID-19 patients of Case study 1, including information about their presence in Mick et al models.

| **Feature** | **Description** | **Present** | **Pathway** |
| --- | --- | --- | --- |
| IFI27 | Interferon Alpha Inducible Protein 27 | **Yes** | Interferon gamma signaling and Innate Immune System |
| TRO | Trophinin | **Yes** | Cell signaling during embryo implantation |
| GABARAP | GABA Type A Receptor-Associated Protein | No | Microtubule binding and GABA receptor binding |
| TIMP1 | TIMP Metallopeptidase Inhibitor 1 | **Yes** | G-protein signaling Ras family GTPases in kinase cascades (scheme) and Degradation of the extracellular matrix |
| TPD52L2 | Tumor Protein D52-Like 2 | No | Protein heterodimerization activity |
| RALGDS | Ral Guanine Nucleotide Dissociation Stimulator | No | Phospholipase D signaling pathway and Signaling by GPCR |
| TOLLIP | Toll Interacting Protein | No | Bacterial infections in CF airways and NF-KappaB Family Pathway |
| INMT | Indolethylamine N-Methyltransferase | No | Tryptophan metabolism and Viral mRNA Translation |
| FAM83A | Family with sequence similarity 83, member A | **Yes** | Epidermal growth factor receptor/EGFR signaling pathway |

**Supplementary Table 4** The 14 features of the two 13-feature signatures of the subsampled 35787-feature transcriptomic data between COVID-19 patients and non-COVID-19 patients of Case study 2. (N/A: Non-Available)

| **Feature** | **Description** | **Pathway** |
| --- | --- | --- |
| RPLP1 | Ribosomal Protein Lateral Stalk Subunit P1 | rRNA processing in the nucleus and cytosol and Viral mRNA Translation |
| CXCL10 | C-X-C Motif Chemokine Ligand 10 | Signaling by GPCR and PEDF Induced Signaling |
| CXCL9 | C-X-C Motif Chemokine Ligand 9 | Signaling by GPCR and PEDF Induced Signaling |
| AL022578.1 | N/A | N/A |
| PRR7 | Proline Rich 7, Synaptic | NMDA receptor-mediated excitotoxicity |
| ITPRID1 | ITPR Interacting Domain Containing 1 | Signaling receptor binding |
| CXCR2P1 | C-X-C Motif Chemokine Receptor 2 Pseudogene 1 | N/A |
| IGFBP2 | Insulin Like Growth Factor Binding Protein 2 | Hematopoietic Stem Cells and Lineage-specific Markers and Myometrial Relaxation and Contraction Pathways |
| PCSK5 | Proprotein Convertase Subtilisin/Kexin Type 5 | Lipoprotein metabolism and Signaling by GPCR |
| AC134669.1 | N/A | N/A |
| ROCK1P1 | Rho Associated Coiled-Coil Containing Protein Kinase 1 Pseudogene 1 | N/A |
| EFNA4 | Ephrin A4 | Guidance Cues and Growth Cone Motility and Ras signaling pathway |
| PMP22 | Peripheral Myelin Protein 22 | Neural Crest Differentiation and a6b1 and a6b4 Integrin signaling |
| SPTA1 | Spectrin Alpha, Erythrocytic 1 | RET signaling and Signaling by GPCR |

**Supplementary Table 5** The ten features of the two 9-feature signatures of the original 35787-feature transcriptomic data between COVID-19 patients and non-COVID-19 patients of Case study 2. (N/A: Non- Available)

| **Feature** | **Description** | **Pathway** |
| --- | --- | --- |
| RPLP1 | Ribosomal Protein Lateral Stalk Subunit P1 | rRNA processing in the nucleus and cytosol and Viral mRNA Translation |
| CXCL9 | C-X-C Motif Chemokine Ligand 9 | Signaling by GPCR and PEDF Induced Signaling |
| PRR7 | Proline Rich 7, Synaptic | NMDA receptor-mediated excitotoxicity |
| AL0225578.1 | N/A | N/A |
| CXCL10 | C-X-C Motif Chemokine Ligand 10 | Signaling by GPCR and PEDF Induced Signaling |
| PCSK5 | Proprotein Convertase Subtilisin/Kexin Type 5 | Lipoprotein metabolism and Signaling by GPCR |
| ROCK1P1 | Rho Associated Coiled-Coil Containing Protein Kinase 1 Pseudogene 1 | N/A |
| EFNA4 | Ephrin A4 | Guidance Cues and Growth Cone Motility and Ras signaling pathway |
| NDUFV1 | NADH:Ubiquinone Oxidoreductase Core Subunit V1 | GABAergic synapse and Respiratory electron transport |
| PMP22 | Peripheral Myelin Protein 22 | Neural Crest Differentiation and a6b1 and a6b4 Integrin signaling |

**Supplementary Table 6** Algorithms used for AutoML in JADBio v1.2.16

| **Algorithm** | **Used for** | **Reference** |
| --- | --- | --- |
| Cross-validation | Performance estimation | in-house implementation,^36^ |
| BBC-CV | Performance correction | in-house implementation,^36^ |
| LASSO | (single) Feature Selection | glmnet library, ^42^ |
| SES | (multiple) Feature Selection | in-house implementation,^39^ |
| Decision Trees | Predictive modeling | in-house implementation, ^43^ |
| Ridge Regression | Predictive modeling | in-house implementation, ^44^ |
| Random Forests | Predictive modeling | in-house implementation, ^45^ |
| Support Vector Machines | Predictive modeling | libsvm library, ^46^ |
| COX regression | Predictive modeling | in-house implementation, ^47^ |
| Random survival forests | Predictive modeling | in-house implementation, ^48^ |

**
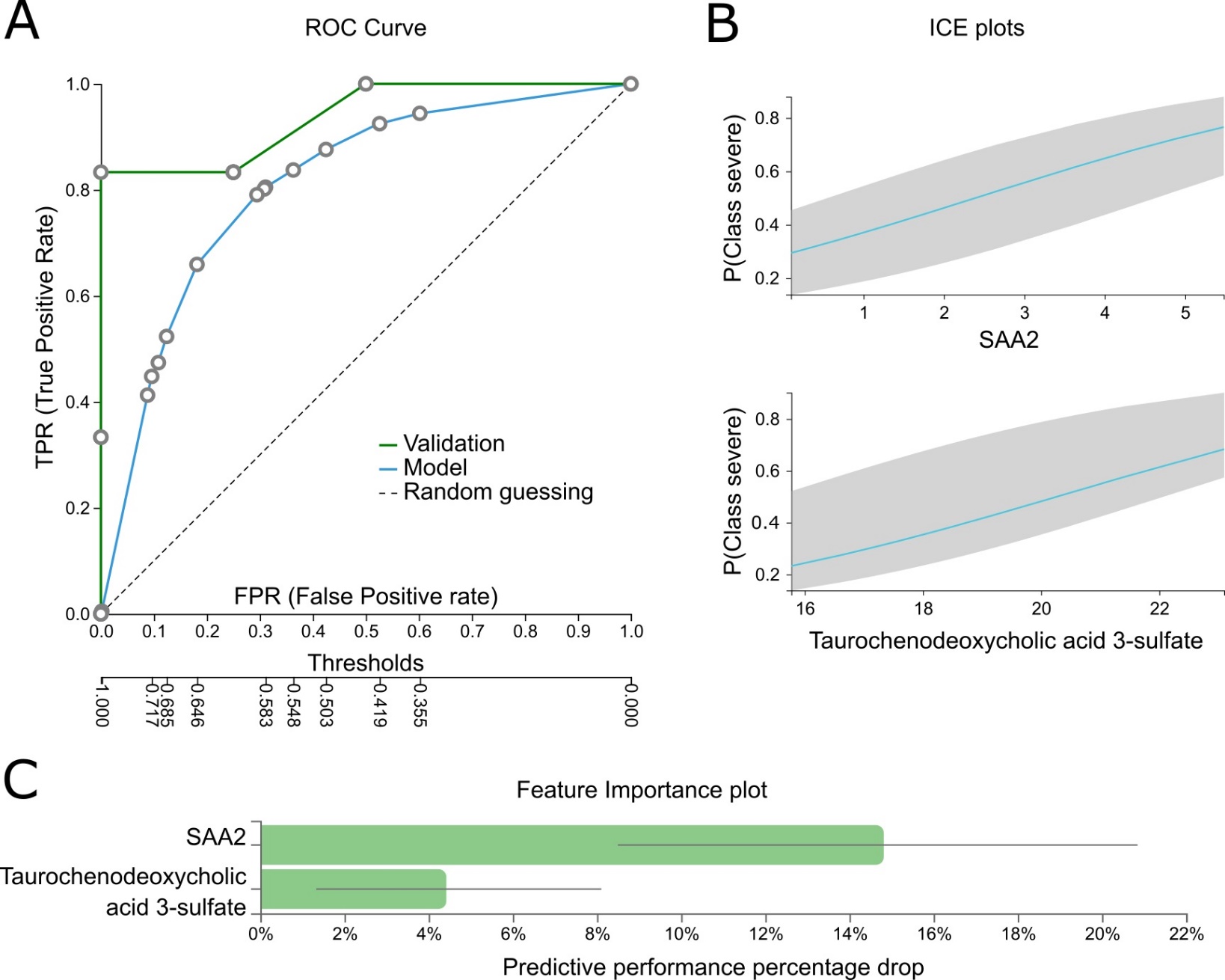
**

**Supplementary Figure 1** *Predictive modeling results of JADBio using aggressive feature selection on the training and validation cohort data of Principal case (Shen et al.).* (A) ROC plot of the model whose signature consists of SAA2 and Taurochenodeoxycholic acid 3-sulfate showing both the training (blue) and validation (green) ROC curves (AUC = 0.958). (B) ICE plots of the selected features indicating the average probability predicted for the severe patients’ class given the value of the markers. (C) Feature importance for SAA2 and Taurochenodeoxycholic acid 3-sulfate defined as the percentage drop in predictive performance when the feature is removed from the model. The grey lines indicate the 95% confidence intervals.
